# A Robust Method for Sample Preparation of Gastrointestinal Stromal Tumour for LC/MS Untargeted Metabolomics

**DOI:** 10.3390/metabo11080554

**Published:** 2021-08-21

**Authors:** Szymon Macioszek, Danuta Dudzik, Julia Jacyna, Agnieszka Wozniak, Patrick Schöffski, Michał J. Markuszewski

**Affiliations:** 1 Department of Biopharmaceutics and Pharmacodynamics, Medical University of Gdańsk, Hallera 107, 80-416 Gdańsk, Poland; szymon.macioszek@gumed.edu.pl (S.M.); danuta.dudzik@gumed.edu.pl (D.D.); julia.jacyna@gumed.edu.pl (J.J.); 2 Department of General Medical Oncology, Leuven Cancer Institute, University Hospitals Leuven, 3000 Leuven, Belgium; agnieszka.wozniak@kuleuven.be (A.W.); patrick.schoffski@uzleuven.be (P.S.); 3 Laboratory of Experimental Oncology, Department of Oncology, KU Leuven, 3000 Leuven, Belgium

**Keywords:** gastrointestinal stromal tumors (GIST), metabolomics, lipidomics, sample preparation, extraction methods, MTBE, Plackett–Burman, DOE

## Abstract

Gastrointestinal stromal tumour has already been well explored at the genome level; however, little is known about metabolic processes occurring in the sarcoma. Sample preparation is a crucial step in untargeted metabolomics workflow, highly affecting the metabolome coverage and the quality of the results. In this study, four liquid-liquid extraction methods for the isolation of endogenous compounds from gastrointestinal stromal tumours were compared and evaluated. The protocols covered two-step or stepwise extraction with methyl-*tert*-butyl ether (MTBE) or dichloromethane. The extracts were subjected to LC-MS analysis by the application of reversed-phase and hydrophilic interaction liquid chromatography to enable the separation and detection of both polar and nonpolar analytes. The extraction methods were compared in terms of efficiency (total number of detected metabolites) and reproducibility. The method was based on the stepwise extraction with MTBE, methanol, and water proved to be the most reproducible, and thus, its robustness to fluctuations in experimental conditions was assessed employing Plackett–Burman design and hierarchical modelling. While most studied factors had no effect on the metabolite abundance, the highest coefficient value was observed for the volume of MTBE added during extraction. Herein, we demonstrate the application and the feasibility of the selected protocol for the analysis of gastrointestinal stromal tumour samples. The method selected could be considered as a reference for the best characterization of underlying molecular changes associated with complex tissue extracts of GIST.

## 1. Introduction

Tumour metabolomics provides an invaluable tool for understanding complex diseases, better patient stratification, prognostics, monitoring disease progression, and therapeutic targets development [1,2,3]. In molecular phenotyping, the broadest possible metabolite coverage is a key player for a comprehensive view and further investigation of the underlying biological condition [4,5]. The great technical advances in analytical chemistry, especially in terms of mass spectrometry, improve its performance in the detection and high-throughput characterization of small molecules [6]. However, more attention should be placed on the selection of the most appropriate and robust sample preparation protocol, particularly in the case of demanding specimens such as tumours tissue. Unfortunately, this key issue is yet often overlooked and should be rather considered as an essential fit-for-purpose step of the metabolomics workflow. Tumour tissue is a specific sample that is usually limited by its small volume, obtained during biopsy, surgery or originating from an animal model. In addition, it is characterised by high cellular heterogeneity or contamination, attributed to remaining blood or healthy tissue [7]. Therefore, both sample harvesting and preparation are critical steps to retrieve the true biological information that allows drawing an accurate picture of the current metabolic status of the studied system [8]. Rapid sampling, including washing step, performed to remove residual blood and immediate metabolic quenching (usually liquid nitrogen −195.8 °C) for complete inactivation of enzymatic activity are crucial to ensure the quality of the studied sample and reliability of the results. Concerning metabolite extraction, mechanical disruption and homogenization are usually necessary to enable effective access of the extracting solvent and hence, efficient extraction of the metabolites from the intracellular compartment [9]. According to their lipophilicity, commonly referred to the LogP, polar (LogP < 0) or medium polar (0 < LogP < 5) compounds can be extracted with the use of liquid-liquid extraction (LLE) and cold mixtures (−20 °C) of organic solvents e.g., methanol (MeOH), ethanol (EtOH), acetonitrile (ACN) or acetone [4]. Nevertheless, non-polar metabolites (LogP > 5) needs non-polar solvent mixtures especially for the extraction of hydrophobically bounded lipids e.g., Folch or Bligh–Dyer methods based on chloroform (CHCl_3_):MeOH:H_2_O [10,11] or as an alternative method with methyl-*tert*-butyl ether (MTBE) extraction (MTBE:MeOH:H_2_O) [12]. Importantly, the Matyash method provides several advantages in terms of extraction efficiency for both polar and non-polar metabolites [13] and safety, giving an option to replace chloroform or dichloromethane, which is particularly relevant due to their high toxicity to humans and the environment [14]. The additional advantage is that MTBE extraction results in the formation of the lipid-rich organic layer on top of the aqueous layer with the protein at the bottom, thus minimizing possible sample contamination [14,15]. Several extraction methods including not only the abovementioned solvents but also other alternatives e.g., sequential butanol (BuOH):MeOH (BUME), heptane:ethyl acetate (EtOAc), the acetic acid method [16], or MeOH:H_2_O followed by organic dichloromethane extraction [17] were explored and compared in different experimental settings [15,18,19,20]. As a separation technique, liquid chromatography (LC) is widely used in untargeted metabolomics [21,22]. LC enables the analysis of a wide range of analytes by using specific chromatographic conditions such as RP or HILIC, which undoubtedly increase metabolome coverage. Besides, it does not require extensive sample preparation. LC, and thus non-complex sample preparation or the ease to hyphenate LC hyphenated to mass spectrometry offers a substantial improvement in the sensitivity and accuracy of the analysis. In addition, LC is a ubiquitous technique so that numerous operators are qualified to operate an LC system, and consumables or accessories are widely available.

**Figure 1:**
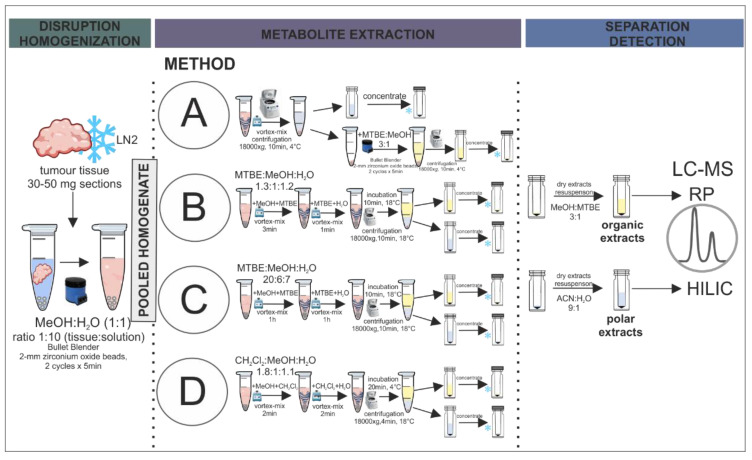
Schematic presentation of the step-by-step workflow of the selected extraction protocols evaluated in the study.

Since extraction significantly affects the captured metabolome, we evaluated different experimental approaches for obtaining meaningful snapshots of the biological state in sarcoma tumours. In our study, the most important aspect was to evaluate extraction methods aiming at the analysis of polar and nonpolar compounds by the application of liquid chromatography-mass spectrometry (LC-MS), in terms of their usefulness for GIST analysis. To extend the metabolite coverage, the separation was performed in two different chromatographic conditions: (i) hydrophilic interaction (HILIC) and (ii) reversed-phase (RP) liquid chromatography, analysed in positive and negative ionisation modes. The general overview of the methodology is presented in Figure 1.

Besides metabolome coverage of the selected method, a great emphasis was placed on its robustness, which ensures that observed results are only due to biological, not analytical variations. In analytical chemistry, method robustness means the ability to maintain consistency after small changes in experimental conditions are introduced [23]. In other words, it says how reproducible the method remains under real-life changes, e.g., in incubation temperatures or reagent concentrations occurring usually by chance in the laboratory environment. When the method is robust, undesired fluctuations of one or more parameters of the established procedure do not significantly impact the results. The evaluation of selected method parameters can be performed with two main methodologies: one-variable-at-a-time (OVAT) approach or design of experiments (DOE). OVAT assumes that only one studied factor can be changed at a time, while other factors need to be maintained at a constant level. Therefore, OVAT is advised to be used only for noncomplex processes. DOE, thanks to the simultaneous changing of a few studied factors, enables a more thorough insight into the studied process. Statistical calculations verify if the method is robust in the studied range and help to find critical steps in the tested procedure [24,25,26].

As above mentioned, the DOE methodology brings more benefits in robustness testing than the OVAT approach. First, fewer experiments are conducted to acquire the same or a larger amount of information. An experiment planned in accordance with the DOE methodology enables one to observe and calculate the impact of each factor on the selected response as well as the interactions between the factors, which is not possible in the OVAT approach. In the case of robustness testing, usually, numerous factors are examined; hence, the screening plans are applied, mainly Plackett–Burman design [23,27]. In a Plackett–Burman matrix, the factors are set at two levels: −1 and +1, but centre points can also be added. For n−1 factors, n experiments have to be performed, while n must be a multiple of 4. The main limitation of Plackett–Burman design is the lack of possibility to explain interactions between the factors, so it must be assumed that all interactions are negligible.

In the present study, the gastrointestinal stromal tumour was the research subject. Despite a better understanding of genome and transcriptome aberrations in GIST cells [28,29,30], there is still a lack of knowledge on metabolic processes characteristic for GIST development. Therefore, the study goal was the selection and evaluation of the optimal sample preparation protocol for further characterization of GIST with the mass spectrometry-based metabolomics approach.

## 2. Results

### 2.1. Extraction Method Development

All extraction procedures were tested on homogenates prepared with the protocol established according to our preliminary observations. The use of five 2 mm beads resulted in satisfactory tissue fragmentation. Moreover, 50% methanol in water yielded more efficient homogenization than 75% methanol in water and pure water. Hence, this homogenization procedure was followed in further assessment of metabolite extraction methods.

**Figure 2:**
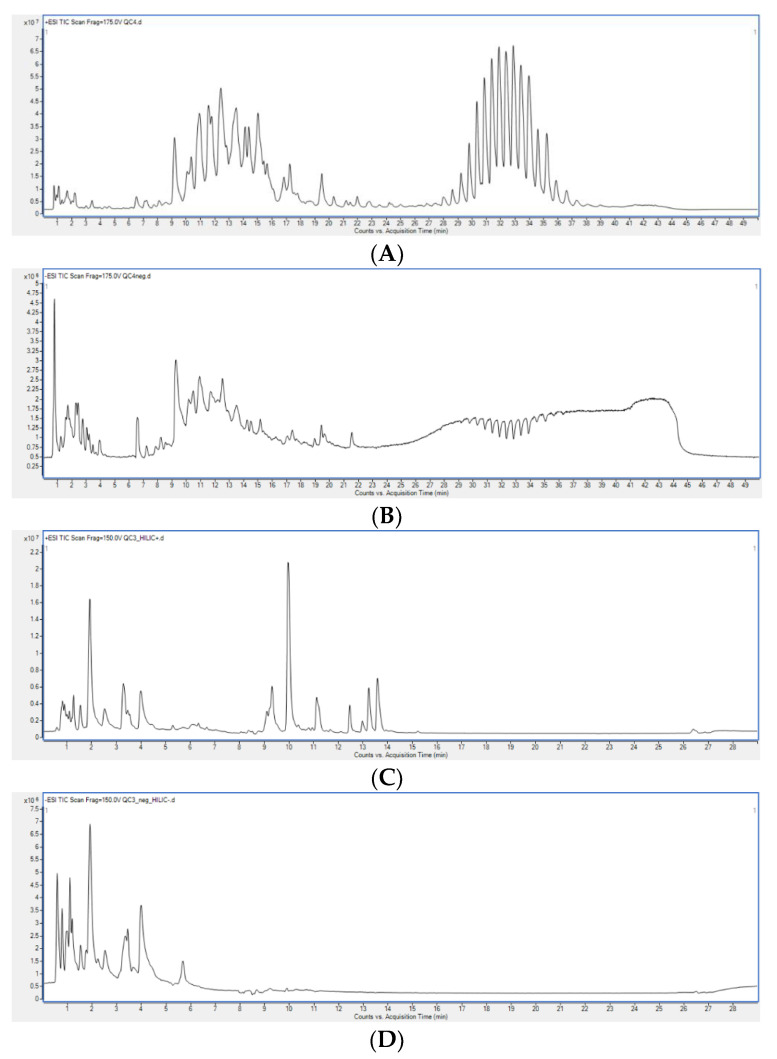
Chromatograms of exemplary samples obtained during (**A**) RP-LC-MS (+), (**B**) RP-LC-MS(-), (**C**) HILIC-LC-MS(+), (**D**) HILIC-LC-MS(-) analyses.

**Figure 3:**
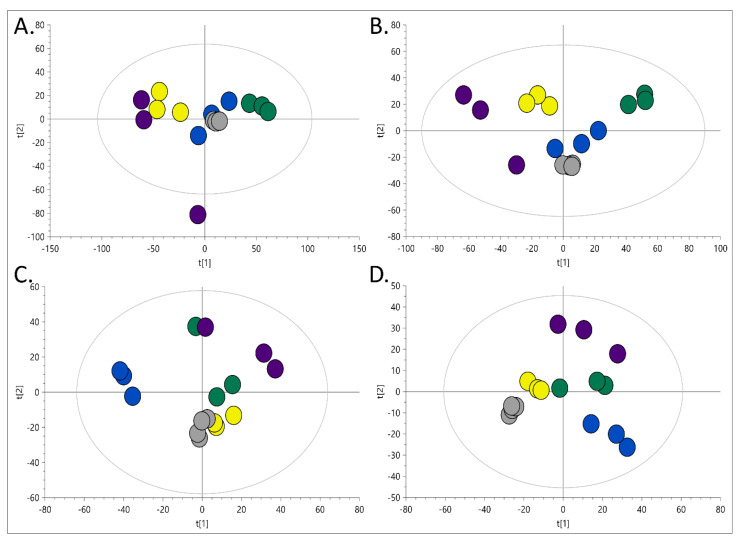
Multivariate PCA-X models built on data acquired during (**A**) RP-LC-MS (+), (**B**) RP-LC-MS(-), (**C**) HILIC-LC-MS(+), (**D**) HILIC-LC-MS(-) analyses. The dots colour indicate the extraction model applied: blue—Method A, yellow—Method B, green—Method C, velvet—method D. The grey dots indicate several injections of the same sample—performed to evaluate apparatus-related variability.

Organic and polar extracts obtained with different extraction methods were analysed using LC-MS. Exemplary chromatograms obtained during the analysis of the studied samples are presented in Figure 2. Subsequently, PCA models were built for each dataset (RP-LC-MS(+), RP-LC-MS(-), HILIC-LC-MS(+), HILIC-LC-MS(-)), which verified the constancy of LC-MS conditions by clustering of the replicates injected from the same vial, and proved higher variability between extraction methods than among replicate samples (Figure 3). 

#### 2.1.1. Organic Extracts Comparison

**Figure 4:**
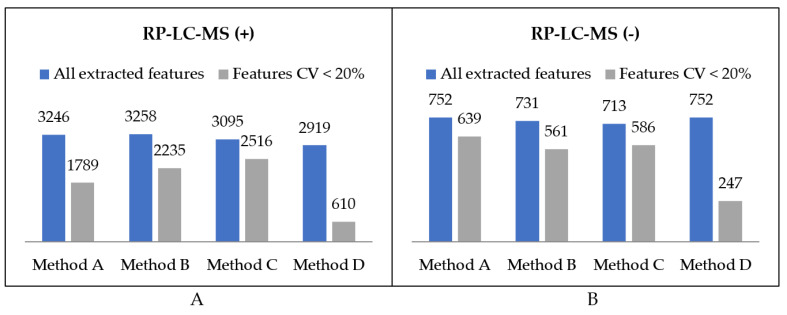
Summary of the number of features detected in organic extracts using RP-LC-MS in (**A**) positive and (**B**) negative ionization modes.

**Figure 5:**
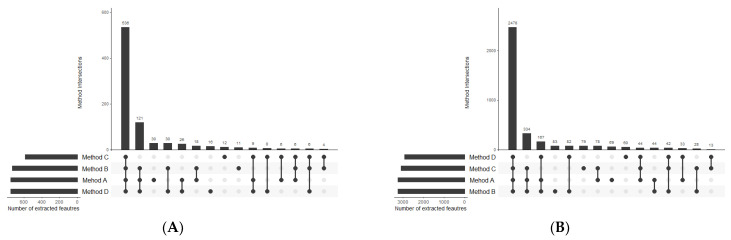
UpSet plots presenting the overlap between the compared extraction methods in terms of lipid compounds detected in (**A**) positive and (**B**) negative ionization modes.

Extraction methods were compared in terms of the total number of metabolic features detected as well as the procedure reproducibility assessed based on the injection of replicate samples. A metabolic feature was assigned as extracted by a method only if it was detected in all three replicates. The numbers of variables detected in organic extracts in positive and negative ionization modes are summarised in Figure 4. Moreover, metabolic features with a coefficient of variation lower than 20% were distinguished. The number of overlapping compounds in the obtained extracts was visualised using UpSet plots [31] (Figure 5).

#### 2.1.2. Polar Extracts Comparison

**Figure 6:**
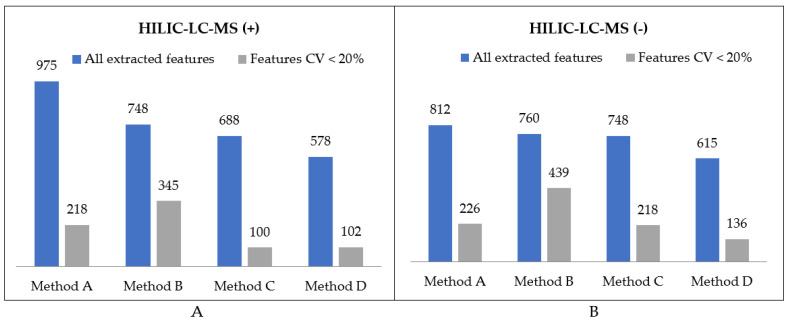
Summary of feature numbers detected in polar extracts using HILIC-LC-MS in (**A**) positive and (**B**) negative MS ionization modes.

**Figure 7:**
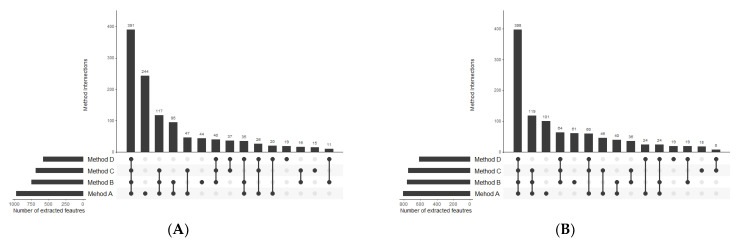
UpSet plots presenting the overlap between compared extraction methods in terms of polar compounds detected in (**A**) positive and (**B**) negative MS ionization modes.

Similarly to the organic extracts, we assessed the extraction of polar metabolites from GIST samples in terms of its efficiency and repeatability. A metabolic feature was assigned as extracted by a method only if it was detected in all three replicates. The corresponding data are presented on the graph in Figure 6. The intersection of metabolites detected with HILIC-LC-MS after extraction with four procedures is shown in Figure 7.

**Figure 8:**
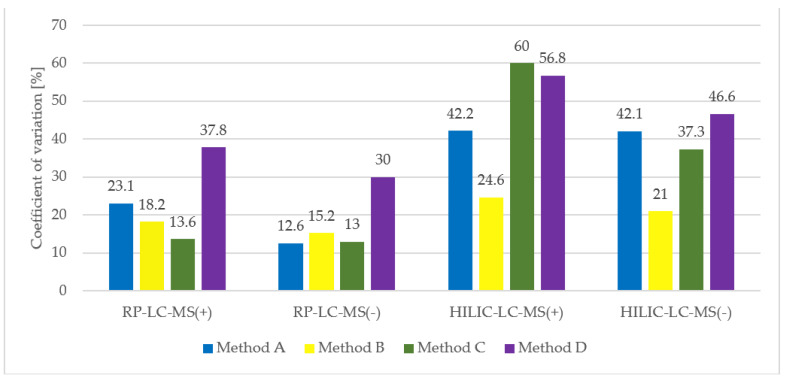
Summary of extraction methods reproducibility based on average CV value of metabolite intensity, calculated based on three replicate extracts.

To summarize the repeatability of the tested procedures, average CV values of all extracted compounds for each procedure were calculated and visualised (Figure 8).

#### 2.1.3. Metabolite Composition of Organic and Polar Fractions

Based on the presented results, method B, relying on two-phase extraction with MTBE, methanol, and water (1.3:1:1.2 *v*/*v*), was chosen as the most appropriate one for untargeted metabolomic analysis of GIST tumour samples. The lipidomic profile obtained using the selected extraction procedure covered several classes of compounds including glycerolipids, ceramides, glycerophosphocholines, glycerophosphoethanolamines, glycerophosphoinositols, sterols, fatty acids, fatty aldehydes, and esters, all detected in positive ionization mode. In turn, negative ionization mode enabled detection of ceramides, glycerophosphoinositols, glycerophosphoglycerols, glycerophosphoethanolamines, glycerophosphocholines, glycerophosphates, glycerolipids, and fatty acids.

In polar extracts, metabolites from various chemical groups and biochemical pathways were determined. In positive ionization mode, a considerable fraction of detected compounds belonged to amino acids and their derivatives. Among other metabolites, numerous purines, pyrimidines, acylcarnitines, and a few medium-chain fatty acids were observed. In negative ionization mode, nucleosides, amino acids and other organic acids, lactones, or vitamins were detected.

### 2.2. Robustness Testing of the Extraction Procedure

The stability of LC-MS conditions during analyses for robustness testing was validated by the injection of quality control (QC) samples (Figure S1). The total number of detected features, excluding the ones found in blank samples, were 1576 for RP-LC-MS (+), 677 for RP-LC-MS ESI (-), 955 features for HILIC-LC-MS (+), and 863 for HILIC-LC-MS (-). PCA score plots for four data matrices are shown in Figure S2.

For each of 10 selected factors (and additional dummy variable), its influence on metabolite abundance was evaluated using hierarchical modelling. Coefficient values (here denoted as beta) indicate the strength of the effect of the factor on the metabolite abundances measured by LC-MS in four sequence runs. The coefficient equal to 0.1 means that the increase in factor value by 1, e.g., from 0 to +1 leads to a 10% increase in metabolite abundance on average. The coefficients for most factors are close to 0; therefore, the factors have no influence on the metabolite abundance. The whole set of model parameters is displayed in the Supplementary Materials (Tables S3–S6).

## 3. Discussion

The overall objective of the study was to develop a GIST sample preparation method for subsequent untargeted metabolomic analysis by means of LC-TOF-MS. So far, in the literature limited information including only one conference abstract concerning GIST tissue metabolomic profiling has been published [32]. However, nuclear magnetic resonance, which requires distinct sample treatment, was used instead of mass spectrometry. With no prior knowledge about GIST tissue composition, several methods were tested, with different solvents and solvent ratios. Our concept assumed avoiding highly toxic chloroform, which is a potent carcinogen as well as a serious threat to the environment. A relatively environment-friendly alternative is MTBE, which nowadays is considered also a standard solvent for lipid extraction [12,33,34,35]. Moreover, dichloromethane was tested, as it is the least toxic simple chlorohydrocarbon. The method optimized by Wang et al., based on the stepwise addition of dichloromethane, methanol, and water, was applied as it was superior to others based on dichloromethane in the case of oesophageal tissue [36]. As expected, dichloromethane posed similar challenges as chloroform. The dichloromethane layer is present at the bottom of the tube; moreover, the tissue debris, proteins, and non-extracted insoluble matrix form a tight layer at the interface, which has to be punctured to collect the lipids. Consequently, in our study, the reproducibility of this method was inferior to methods based on MTBE.

The MTBE-based extraction procedure was first introduced in 2008 by Matyash et al. and has been cited about 1400 times [12]. It was developed for lipid extraction as an alternative to Folch or Bligh and Dyer methods. Its most significant advantage is the formation of a lipid layer as an upper phase due to the low density of MTBE. This leads to faster and cleaner recovery of major lipid classes. However, for the simultaneous extraction of polar metabolites and lipids, a modification to the established Matyash method was made by Sostare et al. [18]. Tested on three different biological matrices, the new method resulted in a higher number of peaks as well as higher metabolite intensities and extraction reproducibility when compared to the original Matyash method and the Bligh and Dyer procedure. Therefore, this modified Matyash procedure was included in our study, together with another method based on the Matyash extraction, applied previously on mouse tissue [37]. To evaluate the differences between stepwise and two-step extraction, another method in this experiment was adapted from Anwar et al. [38].

The first step of optimal method selection was the evaluation of lipid extraction. As far as the total number of extracted lipid features is concerned, there was not a huge difference between the methods, with the negligible advantage of Method A. However, the number of features with abundance CV smaller than 20% visibly distinguish the methods in terms of their reproducibility (Figure 4). While methods A, B, and C enabled the extraction of 2428, 2796, and 2802 lipid features, respectively, with a satisfactory reproducibility, only 857 lipid features were reproducibly isolated with the method based on dichloromethane. This result supports the observations on the hindered collection of non-polar fractions when high-density solvents are used such as chloroform or dichloromethane.

With regards to HILIC separation, in both positive and negative MS modes the highest number of metabolic features was extracted with the two-step MTBE-based method A. Nevertheless, the numbers significantly differ if only reproducible features are concerned. We detected 784 reproducibly extracted features in the polar extracts prepared with method B, compared to 444, 318, and 238 features for methods A, C, and D, respectively. Moreover, a significantly higher reproducibility of method B is reflected in the mean CV value of all extracted features, which for method B is at least twice lower than for other methods. Unexpectedly, the lowest reproducibility characterizes method D, which produces two-phase extracts with the polar layer being the upper one. As the collection of polar phase is noncomplicated, the extraction process itself seems to be of low reproducibility.

For the selection of the optimal extraction procedure, the emphasis was placed on the method reproducibility, rather than the total number of features. Here, the threshold for the CV of signal abundance was set at 20% as it is the most common value for assuring the quality of obtained metabolomics data [39,40,41]. In untargeted metabolomics projects, the metabolic features with higher CV are advised to be filtered out to diminish the noise and facilitate statistical analysis. Otherwise, such unreproducible measurements can result in false positives and, consequently, in irrelevant biological conclusions. Therefore, although method C was the most reproducible in terms of lipid extraction, much larger differences were observed in the reproducibility of polar compounds extraction. As method B extracted polar metabolites in the most reproducible manner, it was chosen as the optimal one for our purpose, overall.

The selectivity of the compared extraction methods was also inspected. In lipid isolation, the vast majority of lipid features was extracted by all four compared methods (Figure Venn). In polar extracts, also the majority of overlapping compounds was covered by all four methods; however, a relatively high number of features were characteristic for methods A, B, and C but not extracted with method D. Moreover, method A is capable of extracting different metabolites than the other methods. In this method, the polar extract is obtained directly from the homogenate as the first step of the procedure, and consequently, it can cover a considerably higher part of the polar metabolome, however, without satisfactory reproducibility.

**Figure 9:**
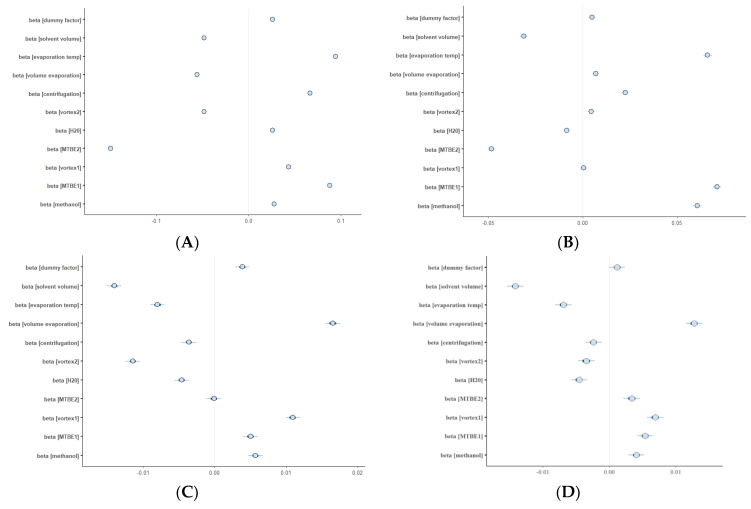
Plots showing the multilevel modelling results. The blue dots represent the coefficient (*beta*) values of 11 factors, together with their standard deviations. The response was the abundance of metabolites detected in (**A**) RP-LC-MS (+), (**B**) RP-LC-MS(-), (**C**) HILIC-LC-MS(+), (**D**) HILIC-LC-MS(-) analyses.

To assure the quality of a metabolomic study employing the developed extraction method, we decided to assess the robustness of the protocol. Robustness testing is not common in untargeted metabolomics; however, it can be useful to identify the critical points of the sample preparation procedure [42,43], as well as of other stages of the metabolomic study pipeline. Indeed, sample preparation highly influences the reliability of metabolite measurements. Most importantly, the sample preparation method has to be reproducible and robust with respect to minor changes in experimental conditions. In the present study, the selected sample extraction method was verified as robust in terms of lipids extraction as well as in the case of polar metabolites. In other words, small fluctuations in pipetted volumes, vortexing, or centrifugation times had no significant effect on the metabolite abundances measured by LC-MS. Hierarchical models built on LC-MS data resulted in the highest values of coefficients in the RP LC-MS (+) dataset (Figure 9). The highest value was obtained for the volume of MTBE added for extraction. The coefficient equal to −0.15 means that a subtle increase in the volume of added MTBE led to an average of 15% decrease in the lipid compounds abundance, possibly caused by higher dilution of the analytes. In conclusion, the MTBE volume can be treated as a critical variable in maximizing the reproducibility of the extraction procedure and it is worth controlling the pipetting process during the sample preparation protocol.

## 4. Materials and Methods

### 4.1. Chemicals and Reagents

MTBE, dichloromethane, and ammonium formate were from Sigma-Aldrich (Saint Louis, MO, USA). Ammonia solution in water (25%) was obtained from Merck KGaA (Darmstadt, Germany). MS additive formic acid (98%) was purchased from Chem-Lab NV (Zedelgem, Belgium). MS-grade isopropanol and acetonitrile were obtained from J. T. Baker (Phillipsburg, NJ, USA), and methanol was from VWR Chemicals (Darmstadt, Germany). Ultrapure water was produced in-house by Direct- Q^®^ 3UV (Millipore, Vienna, Austria). For MS calibration, a low concentration tuning mix was purchased from Agilent Technologies (Santa Clara, CA, USA).

### 4.2. Sample Collection

Samples from mouse xenograft models were obtained with the consent of the Medical Ethics Committee, University Hospitals Leuven (S53483) and the KU Leuven Ethics Committee for Animal Research (project numbers P056/2014, P175/2015). Xenograft models were created through injection of patient-derived tumours into a nude mouse with an inhibited immune system. When passaged to the next mice, the tumours are proved to remain genetically identical to the ones resected from the patients; thus, they can be studied as a substitute for human tissue.

### 4.3. Tissue Sample Homogenization

Samples were stored at −80 °C prior to analysis. After immersing in liquid nitrogen, tumours were cut into 30–50 mg sections and placed in an Eppendorf tube together with five 2 mm diameter zirconium oxide beads. For homogenization, a mixture of methanol and water (1:1, *v*/*v*) was added to the Eppendorf tubes in such quantity that for every 1 mg of tissue, 10 µL of solvent was used. Samples were homogenised in a Bullet Blender tissue homogenizer (Next Advance, Averill Park, NY, USA) in two 5-min cycles at full speed, with samples cooling on ice between the cycles. Then, the obtained homogenates were pooled into one glass tube and vortexed.

### 4.4. Methods for Metabolite Extraction

For metabolite extraction, 500 µL of pooled homogenate was pipetted into glass tubes. Each extraction method was performed on three homogenate replicates to assess method reproducibility. The methods were adapted from already established procedures, retaining the originally applied solvent ratios. A graphical presentation of the applied extraction methods is provided in Figure 1.

Method A, based on Anwar et al. [38], was a two-step extraction in which the polar fraction was obtained through the centrifugation of the homogenate, and the organic fraction was prepared by adding 500 µL of MTBE:methanol mixture (3:1, *v*/*v*) to the homogenised tissue residue. After two cycles of homogenization, the extract was centrifuged and the organic fraction was transferred to a borosilicate glass vial.

Method B was adapted from Sostare et al. [18]. This two-phase extraction protocol started with the addition of 100 µL of methanol and 200 µL of MTBE to the homogenate. After 3 min of vortexing, 320 µL of MTBE and 230 µL of water was added. Then, another 1-min vortexing cycle was performed, and the sample was incubated at 18 °C for 10 min. The tubes were centrifuged at 18,000× *g* at 18 °C for 10 min. From the obtained extract, 200 µL of upper organic fraction and 300 µL of polar bottom fraction were transferred into separate vials.

Method C, based on Chen et al. [37], was a two-phase extraction procedure with MTBE, methanol, and water in the ratio 20:6:7 (*v*/*v*). 50 µL of methanol and 1 mL of MTBE were added to the homogenate, vortexed for 1 h, and 100 µL of water was added. The sample was incubated at 18 °C for 10 min followed by centrifugation at 18,000× *g* at 18 °C for 10 min. Polar and organic fractions (300 µL both) were transferred to LC-MS vials.

Method D was also a two-phase extraction procedure, but here dichloromethane was applied in combination with methanol and water (1.8:1:1.1, *v/v/v*) [36]. The homogenate was vortexed with 160 µL of methanol and 160 µL of dichloromethane for 2 min. Afterwards, another portion of 160 µL of dichloromethane was added together with 170 µL of water and the mixture was again vortexed for 2 min. The next step was sample incubation at 18 °C for 20 min. After centrifugation at 18 °C at 18,000× g for 4 min, 300 µL of polar fraction and 200 µL of organic fraction were transferred to separate vials.

All extracts were vacuum dried at vacuum centrifugal concentrator MiVac DUO (GeneVac, Ipswich, United Kingdom) and the vials were capped. Along with the extracts, blank samples were prepared, where 500 µL of a methanol:water mixture, instead of the homogenate, was subjected to extraction procedures. Prior to the analysis, the organic samples were reconstituted in methanol:MTBE (3:1, *v*/*v*) mixture while the polar samples were in acetonitrile:water (9:1, *v*/*v*).

### 4.5. LC-MS Analysis

The organic extracts consisting mostly of lipid components were analysed by an Agilent 1200 HPLC coupled to 6224 TOF/MS system (Agilent Technologies, Waldbronn, Germany) with a reversed-phase InfinityLab Poroshell 120 EC-C8 column (2.1 mm × 150 mm, 2.7 µm, Agilent Technologies, Santa Clara, CA, USA). For positive ionization mode, phase A was 10 mM ammonium formate water solution (pH = 6.5) and phase B: 10 mM ammonium formate solution in a mixture of methanol and isopropanol (85:15, *v*/*v*, pH = 7.0). For negative ionization mode, phase A was 0.1% formic acid in water and phase B: 0.1% formic acid in a mixture of methanol and isopropanol (85:15, *v*/*v*). The gradient program started at 82% of phase B, increasing to 96% at 30 min when it was held for 8 min. For the next 2 min, 100% of phase B was maintained, and finally, the column was re-equilibrated at initial conditions for 8 min. The injection volume was 5 µL and the flow rate was 0.5 mL/min. The mass range for detection was 61–1200 *m/z*.

For the separation of polar metabolites, 2.1 mm × 100 mm, 2.7 µm Poroshell 120 HILIC column (Agilent Technologies, Santa Clara, CA, USA) was used. Mobile phases in both ionization modes were 10 mM ammonium formate in water (phase A) at pH 6.5 and acetonitrile (phase B). The gradient started from 5% of phase B, up to 45% of phase B at 22 min. The maximum content of the aqueous phase was held for 1 min, and then, the re-equilibration time at initial ratios was 5.5 min. The injection volume for polar extract was 2 µL and the flow rate was 0.4 mL/min. MS parameters are presented in Supplementary Table S1.

A replicate sample, which was a pool of equal volumes of organic or polar extracts, was injected every four samples of the analytical batch to control the stability and repeatability of the analytical procedure.

### 4.6. Data Processing

Raw data were imported into MassHunter Profinder B.08.00 (Agilent Technologies, Santa Clara, CA, USA) for peak deconvolution and time alignment. For the data obtained in positive ionization mode, adducts with H^+^, Na^+^, K^+^, NH_4_^+^ were selected as well as neutral loss of water. For negative ionization mode, adducts with H^−^, HCOO^−^, Cl^−^ and the loss of a water molecule were considered. All MS signals lower than 200 counts were considered background noise. For peak alignment between samples, a retention time shift of 0.15 min was allowed as well as a 15 ppm error for mass detection. Afterwards, the data were filtered to retain only the features present in all replicates for each extraction method. Moreover, features detected in blank samples were discarded. The metabolites annotation was carried out using online databases such as LIPID MAPS, METLIN, and Human Metabolome Database through the CEU Mass Mediator platform (http://ceumass.eps.uspceu.es/ (accessed on 20 May 2021)) [44].

### 4.7. Robustness Testing of the Selected Extraction Method

Extraction procedure robustness was assessed using the Plackett–Burman experimental design. Ten factors were selected as possibly affecting the method robustness. The established factor levels are expected to reflect possible fluctuations during sample preparation in a metabolomics project. In Plackett–Burman design, twelve experiments are required for the evaluation of ten factors. One additional dummy variable was added as an estimate of standard error. Moreover, three replicates at centre points were included to control measurement uncertainty. The experimental matrix is given in Table 1. Fifteen samples were prepared from a pooled homogenate according to the conditions in Table 1. Similar to the method development process, blank samples were also analysed to filter out features originating from solvents or reagents. A QC sample was injected from the same vial throughout the analytical batch to control the signal drift.

The samples prepared according to the experimental matrix were analysed using the same methods as previously described in Section 4.5. The signals were aligned, filtered, and transformed using the logarithm function. The relationship between each tested factor and metabolite abundances was calculated by hierarchical linear modelling using the cmdstanr package in R Studio software (version 1.4.1106). This methodology has recently been applied by our research group under the supervision of Julia Jacyna. First, the models included between-analyte variability in responses to the factors; however, as metabolites generally demonstrated a similar trend, the models were simplified. The advantage of using hierarchical modelling instead of numerous simple linear regression models is the lack of multiple comparisons problem. Moreover, hierarchical models enable information sharing between metabolites and model generalization to other metabolites.

## 5. Conclusions

Various tissues specimens may represent different metabolome compositions. Therefore, the most appropriate sample preparation method should be carefully selected. In this study, four metabolite extraction protocols were compared in terms of extraction efficiency and metabolite isolation reproducibility. The selected method, based on stepwise extraction with MTBE, methanol, and water (1.3:1:1.2, *v*/*v*) was subjected to robustness testing to maximise the quality of the data obtained during untargeted metabolomics analysis. The study proves the usefulness of the DOE approach and multilevel modelling for the assessment of extraction method robustness. Although not common, we suggest the methodology be implemented more often during the selection of a sample preparation protocol as one of the quality assurance steps.

## Figures and Tables

**Table 1:** Plackett–Burman experimental matrix.

	Pattern	Metanol [µL]	Mtbe1 [µL]	vortex1 [min]	MTBE2 [µL]	H20 [µL]	Vortex2 [min]	Centrifugation [min]	Volume for Evaporation [µL]	Evaporation Temperature [°C]	Solvent Volume for Reconstitution [µL]	Dummy Factor
1	+−−−+−−+−++	102	196	165	315	234	50	9	204	33	204	1
2	+++−−−+−−+−	102	204	195	315	226	50	11	196	33	204	−1
3	−−+−+++−−−+	98	196	195	315	234	70	11	196	33	196	1
4	−+−−+−+++−−	98	204	165	315	234	50	11	204	37	196	−1
5	−−−+−−+−+++	98	196	165	325	226	50	11	196	37	204	1
6	0	100	200	180	320	230	60	10	200	35	200	0
7	+−+++−−−+−−	102	196	195	325	234	50	9	196	37	196	−1
8	−+++−−−+−−+	98	204	195	325	226	50	9	204	33	196	1
9	−−+−−+−+++−	98	196	195	315	226	70	9	204	37	204	−1
10	0	100	200	180	320	230	60	10	200	35	200	0
11	++−−−+−−+−+	102	204	165	315	226	70	9	196	37	196	1
12	+++++++++++	102	204	195	325	234	70	11	204	37	204	1
13	+−−+−+++−−−	102	196	165	325	226	70	11	204	33	196	−1
14	−+−+++−−−+−	98	204	165	325	234	70	9	196	33	204	−1
15	0	100	200	180	320	230	60	10	200	35	200	0

## Data Availability

The data presented in this study are available on request from the corresponding author for privacy.

## Supplementary Materials

The following are available online at https://www.mdpi.com/article/10.3390/metabo11080554/s1 Figure S1: Chromatograms of four quality control samples analysed with the use of A) RP-LC-MS ESI (+), B) RP-LC-MS ESI (-), C) HILIC-LC-MS ESI (+), D) HILIC-LC-MS ESI (-), Table S1: Mass spectrometer parameters during the analysis of lipid (RP) and polar (HILIC) extracts, Table S2: Comparison of extraction methods: the average number of extracted metabolites in three replicates, standard deviation, and number of metabolites common to all three replicates, Figure S2: PCA models built on datasets obtained during robustness testing using the following techniques: (a) RP-LC-MS (+), (b) RP-LC-MS(-), (c) HILIC-LC-MS(+), (d) HILIC-LC-MS(-), Table S3: Parameters of hierarchical models developed for RP-LC-MS (+) robustness testing data, Table S4: Parameters of hierarchical models developed for RP-LC-MS (-) robustness testing data, Table S5: Parameters of hierarchical models developed for HILIC-LC-MS (+) robustness testing data, Table S6. Parameters of hierarchical models developed for HILIC-LC-MS (-) robustness testing data.
