# Supplementary material for: A Robust Method for Sample Preparation of Gastrointestinal Stromal Tumour for LC/MS Untargeted Metabolomics
Source: Metabolites. 2021 Aug 21;11(8):554. doi: 10.3390/metabo11080554 (PMC8400919; doi:10.3390/metabo11080554)
Supplement: Supplementary file 1 [file metabolites-11-00554-s001.zip › metabolites-1332441-supplementary.pdf]

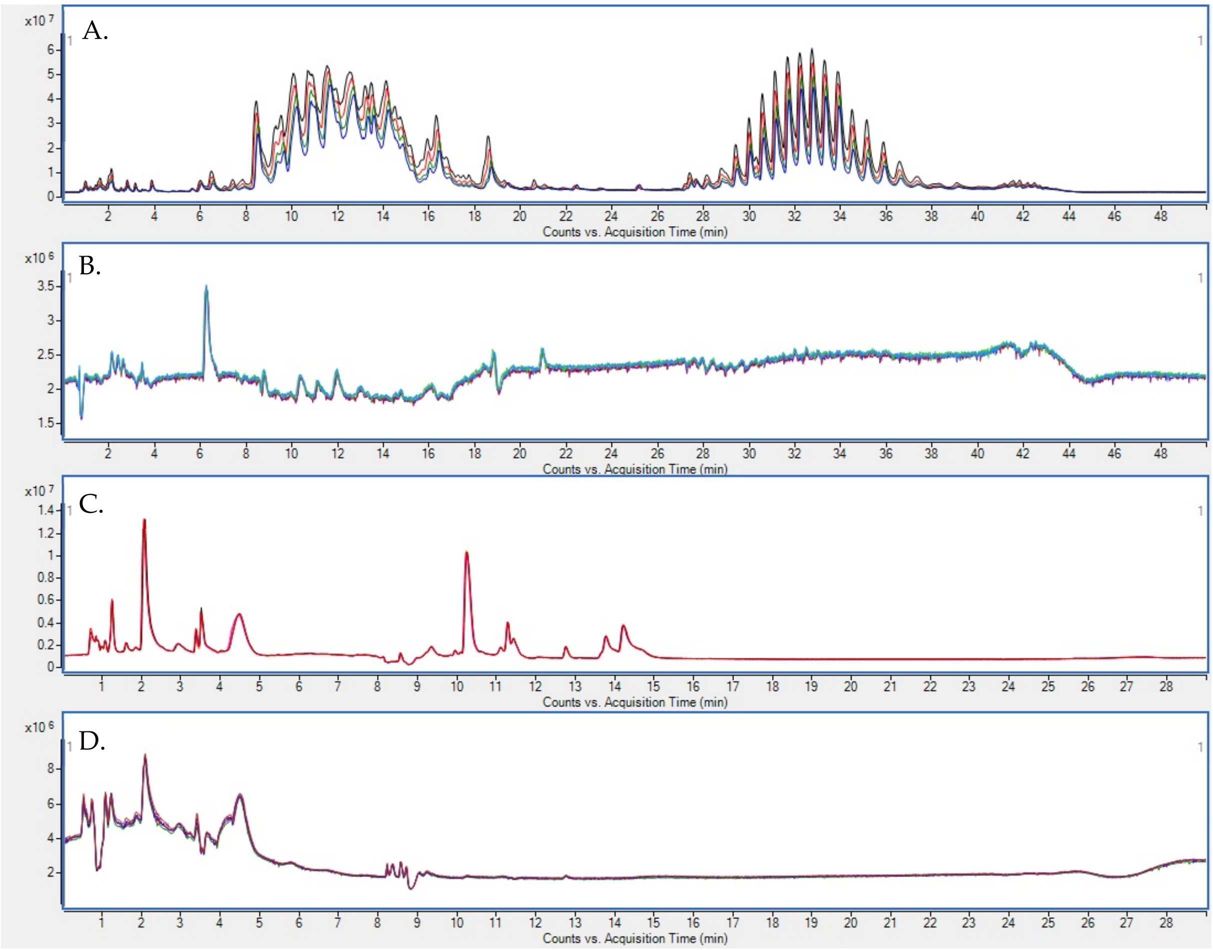

Figure S1. Chromatograms of four quality control samples analysed with the use of A) RP-LC-MS ESI (+), B) RP-LC-MS ESI (-), C) HILIC-LC-MS ESI (+), D) HILIC-LC-MS ESI (-).

Table S1. Mass spectrometer parameters during the analysis of lipid (RP) and polar (HILIC) extracts.

Parameter	***RP***	***HILIC***
Gas temperature	*350 ºC*	*350 ºC*
Gas flow	*10 l/min*	*11 l/min*
Nebulizer pressure	*45 psi*	*45 psi*
Fragmentor voltage	*175 V*	*150 V*
Skimmer voltage	*65 V*	*65 V*

Table S2. Comparison of extraction methods: the average number of extracted metabolites in three replicates, standard deviation, and number of metabolites common to all three replicates.

Technique	Extraction method	Average number of extracted features	Standard deviation	Number of common features (3/3 replicates)	% of common features
RP-LC(+)	Method A	3462	21	3246	94%
	Method B	3448	8	3258	94%
	Method C	3328	41	3095	93%
	Method D	3323	85	2919	88%
RP-LC(-)	Method A	796	17	752	94%
	Method B	793	7	731	92%
	Method C	766	16	713	93%
	Method D	809	14	752	93%
HILIC(+)	Method A	1062	5	975	92%
	Method B	850	14	748	88%
	Method C	889	31	688	77%
	Method D	773	88	578	75%
HILIC(-)	Method A	916	58	812	89%
	Method B	842	29	760	90%
	Method C	882	19	748	85%
	Method D	784	23	615	78%

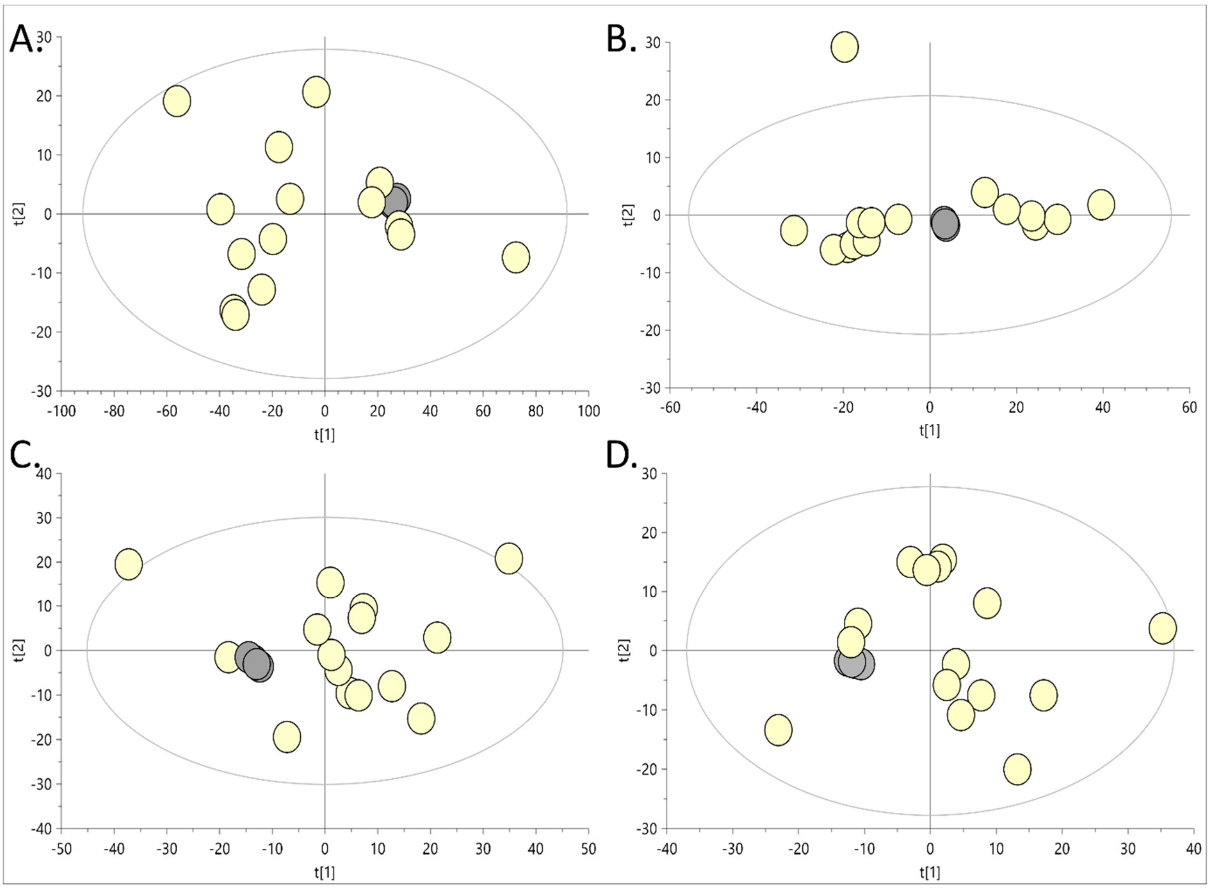

Figure S2. PCA models built on datasets obtained during robustness testing using the following techniques: (a) RP-LC-MS (+), (b) RP-LC-MS(-), (c) HILIC-LC-MS(+), (d) HILIC-LC-MS(-). Grey dots correspond to quality control samples while yellow dots represent experimental design samples.

Table S3. Parameters of hierarchical models developed for RP-LC-MS (+) robustness testing data, mean – mean coefficient sd – standard deviation of the coefficient, mad – median absolute deviation, q5 – 5 percentile, q95 – 95 percentile, rhat – rhat statistics.

Factor	mean	median	sd	mad	q5	q95	rhat
Methanol volume	0.0166	0.0166	0.000864	0.000858	0.0152	0.018	1.01
MTBE volume 1	0.0503	0.0502	0.000931	0.000916	0.0488	0.0518	1.02
Vortex 1	0.0243	0.0243	0.000886	0.000903	0.0229	0.0258	1.04
MTBE volume 2	-0.087	-0.087	0.00102	0.00102	-0.0887	-0.0854	1.01
Water volume	0.0153	0.0153	0.000893	0.000898	0.0138	0.0167	1.01
Vortex 2	-0.0284	-0.0284	0.000891	0.000905	-0.0299	-0.027	1.01
Centrifugation time	0.038	0.0379	0.000879	0.00088	0.0366	0.0394	1.01
Volume for evaporation	-0.0318	-0.0318	0.000838	0.000852	-0.0331	-0.0304	1.01
Evaporation temperature	0.0532	0.0532	0.000903	0.000888	0.0518	0.0548	1.02
Solvent volume	-0.0268	-0.0267	0.000894	0.00088	-0.0282	-0.0253	1.02
Dummy factor	0.0151	0.0151	0.000865	0.000825	0.0137	0.0165	1.01

Table S4. Parameters of hierarchical models developed for RP-LC-MS (-) robustness testing data, mean – mean coefficient sd – standard deviation of the coefficient, mad – median absolute deviation, q5 – 5 percentile, q95 – 95 percentile, rhat – rhat statistics.

**BETA**	mean	median	sd	mad	q5	q95	rhat
Methanol volume	0.0419	0.042	0.00126	0.00125	3.98E-02	0.044	1
MTBE volume 1	0.0495	0.0495	0.00126	0.00126	4.74E-02	0.0515	1.01
Vortex 1	0.000926	0.000916	0.00112	0.00113	-9.37E-04	0.00273	1.01
MTBE volume 2	-0.0332	-0.0332	0.00116	0.00113	-3.50E-02	-0.0312	1.01
Water volume	-0.00579	-0.00583	0.00112	0.00111	-7.60E-03	-0.00394	1.01
Vortex 2	0.00192	0.00193	0.0011	0.00103	1.11E-04	0.00377	1
Centrifugation time	0.0154	0.0154	0.00112	0.00108	1.36E-02	0.0172	1
Volume for evaporation	0.00472	0.00469	0.00113	0.00116	2.89E-03	0.00653	1.01
Evaporation temperature	0.046	0.046	0.00124	0.00117	4.39E-02	0.048	1
Solvent volume	-0.0212	-0.0212	0.00114	0.00113	-2.31E-02	-0.0193	1
Dummy factor	0.00312	0.00311	0.00111	0.00112	1.31E-03	0.00496	1.01

Table S5. Parameters of hierarchical models developed for HILIC-LC-MS (+) robustness testing data, mean – mean coefficient sd – standard deviation of the coefficient, mad – median absolute deviation, q5 – 5 percentile, q95 – 95 percentile, rhat – rhat statistics.

**BETA**	mean	median	sd	mad	q5	q95	rhat
Methanol volume	3.38E-03	3.37E-03	5.97E-04	6.06E-04	2.41E-03	4.33E-03	1
MTBE volume 1	2.63E-03	2.64E-03	5.96E-04	5.74E-04	1.68E-03	3.62E-03	1
Vortex 1	7.04E-03	7.05E-03	6.18E-04	6.34E-04	6.04E-03	8.03E-03	1
MTBE volume 2	6.81E-05	6.00E-05	6.32E-04	6.29E-04	-9.61E-04	1.11E-03	1
Water volume	-2.82E-03	-2.83E-03	6.10E-04	6.05E-04	-3.80E-03	-1.79E-03	1
Vortex 2	-7.19E-03	-7.18E-03	5.95E-04	5.80E-04	-8.18E-03	-6.18E-03	1
Centrifugation time	-1.94E-03	-1.93E-03	6.19E-04	6.25E-04	-2.97E-03	-9.57E-04	1
Volume for evaporation	1.10E-02	1.10E-02	6.16E-04	6.06E-04	9.97E-03	1.20E-02	1
Evaporation temperature	-5.39E-03	-5.40E-03	6.32E-04	6.49E-04	-6.40E-03	-4.37E-03	1
Solvent volume	-1.00E-02	-1.01E-02	6.28E-04	6.19E-04	-1.11E-02	-9.00E-03	1
Dummy factor	2.89E-03	2.89E-03	5.88E-04	5.59E-04	1.89E-03	3.86E-03	1

Table S6. Parameters of hierarchical models developed for HILIC-LC-MS (-) robustness testing data, mean – mean coefficient sd – standard deviation of the coefficient, mad – median absolute deviation, q5 – 5 percentile, q95 – 95 percentile, rhat – rhat statistics.

**BETA**	mean	median	sd	mad	q5	q95	rhat
Methanol volume	0.00315	0.00315	5.74E-04	5.74E-04	2.21E-03	4.07E-03	1
MTBE volume 1	0.00417	0.00417	5.68E-04	5.62E-04	3.26E-03	5.11E-03	1
Vortex 1	0.00535	0.00534	5.77E-04	5.85E-04	4.38E-03	6.28E-03	1
MTBE volume 2	0.00262	0.00262	5.99E-04	5.98E-04	1.64E-03	3.58E-03	1
Water volume	-0.00352	-0.00352	5.64E-04	5.74E-04	-4.46E-03	-2.60E-03	1
Vortex 2	-0.00269	-0.00269	6.00E-04	6.37E-04	-3.65E-03	-1.71E-03	1
Centrifugation time	-0.00185	-0.00185	5.70E-04	5.70E-04	-2.78E-03	-9.01E-04	1
Volume for evaporation	0.00987	0.00988	5.84E-04	5.87E-04	8.92E-03	1.08E-02	1
Evaporation temperature	-0.00536	-0.00535	5.88E-04	5.82E-04	-6.35E-03	-4.39E-03	1
Solvent volume	-0.011	-0.011	5.77E-04	5.71E-04	-1.19E-02	-1.00E-02	1
Dummy factor	0.000902	0.000894	5.51E-04	5.60E-04	-1.47E-05	1.80E-03	1
